# The impact of creating mathematical formula to predict cardiovascular events in patients with heart failure

**DOI:** 10.1038/s41598-018-22347-0

**Published:** 2018-03-05

**Authors:** Mari Sakamoto, Hiroki Fukuda, Jiyoong Kim, Tomomi Ide, Shintaro Kinugawa, Arata Fukushima, Hiroyuki Tsutsui, Akira Ishii, Shin Ito, Hiroshi Asanuma, Masanori Asakura, Takashi Washio, Masafumi Kitakaze

**Affiliations:** 1 Department of Clinical Research and Development, National Cerebral and Cardiovascular Center, 5-7-1 Fujishirodai, Suita, Osaka Japan; 2 Department of Cell Biology and National Cerebral and Cardiovascular Center, 5-7-1 Fujishirodai, Suita, Osaka Japan; 3 Department of Cardiovascular Medicine, Faculty of Medical Sciences, Kyushu University, 3-1-1 Maidashi, Higashi-ku, Fukuoka, Fukuoka, Japan; 4 Department of Cardiovascular Medicine, Faculty of Medicine and Graduate School of Medicine, Hokkaido University, Kita 14-jo Nishi 5-chome, Kita-ku, Sapporo, Hokkaido Japan; 5 The Institute of Scientific and Industrial Research, Osaka University, 1-1 Yamadaoka, Suita, Osaka, Japan; 6 Department of Internal Medicine, Meiji University of Integrative Medicine, Hiyoshicho Nantan, Kyoto, Japan; 7 Present Address: Hyogo College of Medicine, 1-1 Mukogawacho, Nishinomiya, Hyogo Japan

## Abstract

Since our retrospective study has formed a mathematical formula, *α* = *f*(*x*_1_, …, *x*_252_), where *α* is the probability of cardiovascular events in patients with heart failure (HF) and *x*_1_ is each clinical parameter, we prospectively tested the predictive capability and feasibility of the mathematical formula of cardiovascular events in HF patients. First of all, to create such a mathematical formula using limited number of the parameters to predict the cardiovascular events in HF patients, we retrospectively determined *f*(*x*) that formulates the relationship between the most influential 50 clinical parameters (*x*) among 252 parameters using 167 patients hospitalized due to acute HF; the nonlinear optimization could provide the formula of *α* = *f*(*x*_1_, …, *x*_50_) which fitted the probability of the actual cardiovascular events per day. Secondly, we prospectively examined the predictability of *f*(*x*) in other 213 patients using 50 clinical parameters in 3 hospitals, and we found that the Kaplan–Meier curves using actual and estimated occurrence probabilities of cardiovascular events were closely correlated. We conclude that we created a mathematical formula *f*(*x*) that precisely predicted the occurrence probability of future cardiovascular outcomes of HF patients per day. Mathematical modelling may predict the occurrence probability of cardiovascular events in HF patients.

## Introduction

Heart failure (HF), one of the leading causes of mortality and morbidity worldwide^1^, is the end stage of many cardiovascular diseases. Although the cause of HF is usually unique for each patient, numerous clinical and social factors, including disease severity, treatment protocols, comorbidity, lifestyle and social environment, independently link to the patients’ prognoses^2–6^, implicated in ‘precision medicine’^7^. However, many studies have not considered the relative contribution of such factors and have not examined the contribution of the unexpected and unknown factors to cardiovascular events. Even if the multiple factors are identified as necessary for the occurrence of cardiovascular events, the results are qualitative and not quantitative. Finally, these factors are usually proved retrospectively because researchers usually do not test the reproducibility of the results in the prospective study, which may not lead the definite conclusion for the identified factors. To overcome these limitations, we devised a mathematical formula using all the parameters and factors in the medical records to provide the occurrence probability of cardiovascular events and revealed that more than 250 factors are linked to the occurrence probability of cardiovascular events in patients with HF^8^. However, one might argue that this formula is merely the result of the fitting of the clinical data with the function of occurrence probability of cardiovascular outcomes and that this mathematical formula may not predict the future clinical outcomes such as a law of gravity^9^ -the law of gravity guarantees the time for an object to reach the ground.

To clarify that our mathematical model prospectively provides the probability of cardiovascular events, we devised a mathematical formula using the clinical retrospective data of patients with HF and tested whether this formula can predict the probability of future clinical cardiovascular events per day in patients with HF. If this is proved, we can obtain the formula to predict the occurrence probability of cardiovascular events using many clinical or social parameters beforehand, leading to the precision medicine of HF^10,11^.

## Methods

### Ethics statement

This study was approved by National Cerebral and Cardiovascular Center Research Ethics Committee (M22-49, M24-51). The Committee decided that the acquisition of informed consent from 167 patients was not required according to the Japanese Clinical Research Guideline because this was a retrospective observational study. Instead, we made a public announcement using both internet homepage of our institution and bulletin boards of our out-patient and in-patient clinics in accordance with the request of the Ethics Committee and the Guideline. For the prospective observational study of 213 patients, we obtained written informed consent after the approval of Research Ethics Committees in three institutes of National Cerebral and Cardiovascular Center and Hokkaido and Kyushu Universities. Registration number of the clinical trial is UMIN000018691 at https://upload.umin.ac.jp/cgi-open-bin/ctr/ctr.cgi?function=brows&action=brows&recptno=R000021637&type=summary&language=J.

### Protocols

#### Protocol I: The creation of the mathematical formula using the retrospective data

Since we retrospectively obtained 252 clinical parameters among 402 parameters in 152 patients with acute decompensated HF (ADHF), calculated the formula to provide the probability of cardiovascular events (the hospitalization or death due to HF)^8^ and added 16 patients in the patients’ cohort after sorting the data, we enrolled 167 patients with ADHF admitted between November 2007 and October 2009. We followed up these patients until the time of cardiovascular events or December 2014. The diagnosis of HF was confirmed by an expert team of cardiologists using the Framingham criteria^12^.

Here, we showed how to create the mathematical formula to predict the cardiovascular events in the previous study. First of all, our hypothesis in the previous study is that we can derive a mathematical formula for the estimation of prognosis, i.e., the equation τ = f(x_1_, …, x_p_), where x_1_, …, x_p_ are clinical features and τ represents the day for the cardiovascular event in the patients with HF, and we showed the positive evidence to support such a hypothesis in the previous study. In the present study, we prospectively tested the predictive capability and feasibility of the mathematical formula of cardiovascular events in HF patients to strengthen the feasibility of the creation of the mathematical formula to predict the probability of the cardiovascular events.

Then we explained how we performed to create the mathematical formula of τ = f(x_1_, …, x_p_) in the previous study. Since we obtained 402 parameters at the discharge following the hospitalization due to ADHF from the data of careful history-taking, physical examinations, laboratory tests, chest X-rays, electrocardiograms, complete Doppler echocardiographic studies, coronary angiography, right heart catheterization, cardiac scintigraphy, cardiovascular magnetic resonance, cardiopulmonary exercise testing and polysomnography in patients with HF, we hypothesized that all or some of the parameters influence the time of cardiovascular events to some extents, and we quantitatively assessed the occurrence probability of the cardiovascular events using the probability model based on the Poisson process. Thus, the probability density *p*_*i*_(*t*) for the cardiovascular events of patient i at an elapsed time t after discharge is represented by the following exponential formula:

1 $${p}_{i}(t)=\frac{1}{{\tau }_{i}}\exp (-\frac{t}{{\tau }_{i}})$$

A mean elapsed time *τ*_*i*_ from discharge to the rehospitalization of patient *i* depends on some of the given clinical factors ${X}^{i}=\{{x}_{1}^{i},\ldots ,{x}_{p}^{i}\}$ of the patient, *i.e*., a common subset ${X}_{S}^{i}\subseteq {X}^{i}$ over all patients. The dependency is primarily approximated by the following inverse linear relation:

2 $${\tau }_{i}\cong \frac{1}{\sum _{{x}_{j}^{i}\in {X}_{S}^{i}}{\beta }_{j}{x}_{j}^{i}+\gamma }$$

where the denominator represents the expected frequency of cardiovascular rehospitalization per day, ${X}_{S}^{i}$ is a set of values of the factors in *X*_*S*_ for patient *i*, *β*_*j*_ is the contributing weight of the *j* th factor to the frequency, and *γ* is the intrinsic frequency for any patient. We considered that *τ*_*i*_ of the patients are sampled from a common population distribution *p*_*τ*_(*τ*). Therefore, the total probability distribution of the rehospitalization time *P*(*t*) is expected to be a superposition of Eq. (1) for various *τ* sampled from *p*_*τ*_(*τ*), as follows, where *p*(*t*) is *p*_*i*_(*t*) in Eq. (1) for a general *τ*:

From these two equations we obtained the following equation.

3 $$P(t)={\int }_{0}^{\infty }{p}_{\tau }(\tau )p(t)d\tau ={\int }_{0}^{\infty }{p}_{\tau }(\tau )\frac{1}{\tau }\exp (-\frac{t}{\tau })d\tau $$

Then we used the following natural conjugate prior distribution for the unknown *p*_*τ*_(*τ*):

4 $${p}_{\tau }(\tau )=\frac{{\tau }^{-n}\exp (-1/\tau {\sum }_{i=1}^{n}{\tau }_{i})}{{\int }_{0}^{\infty }{\tau }^{-n}\exp (-1/\tau {\sum }_{i=1}^{n}{\tau }_{i})d\tau }$$

where *τ*_*i*_ is given by the dataset *D*.

After several steps of the manipulation, we finally described the modeling algorithm. First, the value of every factor ${x}_{j}^{i}\in {X}^{i}$ for all patients $i=1,\ldots ,n$ in *D* was normalized to fit into the interval [0, 1] using the maximum and minimum values. This normalization to eliminate differences in the factor scales was necessary to allow for the measurement of the essential contribution of each factor’s variation to *τ*_*i*_. Subsequently, we applied the equations (1) and (2) to the normalized dataset *D*_*N*_ to model the probabilistic rehospitalization process and we determined the model parameters *β*_*j*_ and *γ* in the equation (1) to maximize the following objective function:

5 $$L({\beta }_{1},\ldots ,{\beta }_{p},\gamma )=\,\mathrm{ln}[\prod _{i=1}^{n}(\sum _{j=1}^{p}{\beta }_{j}{x}_{j}^{i}+\gamma )\exp \{-(\sum _{j=1}^{p}{\beta }_{j}{x}_{j}^{i}+\gamma ){\tau }_{i}\}]-\lambda (\sum _{j=1}^{p}|{\beta }_{j}|+|\gamma |)$$

The first term is the log-likelihood of the model consisting of the previous equations over *D*_*N*_. The second term is called an *L*1-regularization term, which penalizes the coefficients of negligible factors by setting them equal to zero when the larger hyper-parameter *λ* eliminates more factors. This term avoids the over-fitting of the model to the dataset by selecting a set of effective factors ${X}_{S}^{i}$ from a given *X*^*i*^. In our study, *λ* is tuned to be 0.02 to maintain the largest value of the equation (5) similarly to the other parameters *β*_*j*_ and *γ*.

To seek the optimum parameter values of *β*_1_, …, *β*_*p*_, *γ* that maximize the objective function *L*(*β*_1_, …, *β*_*p*_, *γ*), we applied a simple greedy hill-climbing algorithm, in which the parameter values are iteratively modified toward their gradient direction $(\partial L/{\beta }_{1},\ldots ,\partial L/{\beta }_{p},\partial L/\gamma )$. When the improvement of *L* becomes nearly negligible, the resulting parameter values are taken as the optima. Because this process depends on the initial values of the parameters, we repeated this optimization 100 times starting with random initial values and selected the result providing the maximum L. This was how we selected 252 influential parameters among the 402 clinical parameters in the previous study^8^.

Then we selected the most influential 50 parameters among 252 parameters and revised the mathematical formula. The 50 most influential parameters in the present study are defined as the clinical parameters with the 50 highest coefficients values shown in the previous manuscript^8^. The number of the 50 is arbitrary and the realistic values to be collected for the prospective study.

#### Protocol II: The prospective study to validate the mathematical formula

We prospectively enrolled 213 patients with ADHF admitted between May 2013 and March 2015 in three different hospitals of National Cerebral and Cardiovascular Center (*n* = 114) and Hokkaido (*n* = 80) and Kyushu Universities (*n* = 19) and followed up these patients until the time of cardiovascular events or the end of April 2016. The timing of patients’ discharge was determined by an expert team of cardiologists in charge of the HF department; discharge was recommended when patients presented no signs of decompensation such as the New York Heart Association (NYHA) Functional Classfication <3, no sign of rales, no galloping rhythm, stable blood pressure and an improvement in renal function due to an optimal treatment that followed international guidelines^13^. Rehospitalization was defined as hospitalization for decompensated HF and cardiovascular death was defined as the death due to the worsening of HF. The primary endpoint was the first cardiovascular event of either rehospitalization or death due to the worsening of HF.

Then we created the mathematical model for the occurrence probability of cardiovascular events. First of all, we assumed that the probability of cardiovascular events per day of patients does not change significantly from its discharge to its cardiovascular events. We defined the mathematical formula to predict the constant occurrence probability of cardiovascular events per day as follows:

6 $$\alpha =f({x}_{1},\ldots ,{x}_{p}|{\boldsymbol{\beta }},c)={{\boldsymbol{\beta }}}^{T}X+c=\sum _{j=1}^{p}{{\beta }}_{j}{x}_{j}+c$$

where *α* is the estimated occurrence probability of cardiovascular events per day for a patient, $X={({x}_{1},\mathrm{...},{x}_{p})}^{T}$ is a clinical feature vector of the patient, ${\boldsymbol{\beta }}={({\beta }_{1},\mathrm{...},{\beta }_{p})}^{T}$ is a weight vector of the features, and *c* is an intercept of *α*. In this study, 50 clinical features, that is, *p* = 50, was used. As any event occurring with a constant probability in a given time period is generated by a Poisson process^14^, cardiovascular events of a patient also occur through this process with its individual *α*. Thus, the probability density for cardiovascular events of a patient at an elapsed time *t* after discharge is represented by the following exponential formula:

7 $$P(t|X;{\boldsymbol{\beta }},c)=\exp (-\alpha t)=\exp \{-({{\boldsymbol{\beta }}}^{T}X+c)t\}=\exp \{-(\sum _{j=1}^{p}{\beta }_{j}{x}_{j}+c)t\}$$

Given a retrospective dataset ${D}_{R}=\{({X}_{i},{t}_{i})|i=1,\mathrm{...},{N}_{R}\}$ where *X*_*i*_ and *t*_*i*_ are the clinical feature vector and the elapsed days at the cardiovascular event from the discharge of a patient *i*, respectively, the expected survival curve of patients in *D*_*R*_ is represented as:

8 $$\begin{array}{c}{P}_{RE}(t|{\boldsymbol{\beta }},c)={\int }_{{D}_{R}}P(t|X;{\boldsymbol{\beta }},c){P}_{RE}(X)dX={\int }_{{D}_{R}}\exp \{-({{\boldsymbol{\beta }}}^{T}X+c)t\}{P}_{RE}(X)dX\\ \quad \quad \quad \quad \,=\frac{1}{{N}_{R}}\sum _{i\in {D}_{R}}\exp \{-({{\beta }}^{T}{X}_{i}+c)\cdot t\}=\frac{1}{{N}_{R}}\sum _{i\in {D}_{R}}\exp \{-(\sum _{j=1}^{p}{{\beta }}_{j}{x}_{ij}+c)\cdot t\}\end{array}$$

where *P*_*RE*_(*X*) is the population distribution of the retrospective dataset *D*_*R*_. *N*_*R*_ is 167 in our case. Conversely, we directly derived the Kaplan–Meier survival curve *P*_*R*_(*t*) using *D*_*R*_ by following a standard procedure^15^. Then, we estimated the best parameter values of ***β*** and *c*, which minimize the following Kullback–Leibler divergence (KL-divergence)^16^. The KL-divergence is a well-known statistical measure to reveal the discrepancy between two probability distributions.

9 $$\begin{array}{rcl}KL({P}_{R},{P}_{RE}|{\boldsymbol{\beta }},c) & = & \int {P}_{R}(t)\{\mathrm{ln}\,{P}_{R}(t)-\,\mathrm{ln}\,{P}_{RE}(t|{\boldsymbol{\beta }},c)\}dX\\  & = & \frac{1}{{N}_{R}}\sum _{i\in {D}_{RR}}\{\mathrm{ln}\,{P}_{R}({t}_{i})-\,\mathrm{ln}\,{P}_{RE}({t}_{i}|{\boldsymbol{\beta }},c)\}\\  & = & \frac{1}{{N}_{R}}\sum _{i\in {D}_{RR}}[\mathrm{ln}\,{P}_{R}({t}_{i})-\,\mathrm{ln}[\frac{1}{{N}_{R}}\sum _{i\text{'}\in {D}_{R}}\exp \{-(\sum _{j=1}^{p}{{\beta }}_{j}{x}_{i^{\prime} j}+c){t}_{i}\}]]\to \,{\rm{\min }}\end{array}$$

where *D*_*RR*_ is a dataset excluding the patients whose observations are censored and, thus, do not have *t*_*i*_ in *D*_*R*_. The parameters ***β*** and *c* minimizing this measure are determined by using the Nelder–Mead method^17^, which is a renowned non-linear optimization algorithm.

We used these estimated parameter values of ***β*** and *c* to predict the survival curve of a given prospective dataset ${D}_{P}=\{({X}_{i},{t}_{i})|i=1,\mathrm{...},{N}_{P}\}$ where *N*_*P*_ is 213 in our case. The predicted survival curve was obtained by substituting the above-mentioned best values of ***β*** and *c* and the clinical feature vectors *X*_*i*_ of patients in *D*_*P*_ to the following ${P}_{PE}(t|{\boldsymbol{\beta }},{\bf{c}})$.

10 $$\begin{array}{c}{P}_{PE}(t|{\boldsymbol{\beta }},c)={\int }_{{D}_{P}}P(t|X;{\boldsymbol{\beta }},c)p(X)dX={\int }_{{D}_{P}}\exp \{-({{\boldsymbol{\beta }}}^{T}X+c)t\}{P}_{P}(X)dX\\ \quad \quad \quad \quad \,=\frac{1}{{N}_{P}}\sum _{i\in {D}_{P}}\exp \{-({\beta }^{T}{X}_{i}+c)\cdot t\}=\frac{1}{{N}_{P}}\sum _{i\in {D}_{P}}\exp \{-(\sum _{j=1}^{p}{{\beta }}_{j}{x}_{ij}+c\cdot t)\}\end{array}$$

We compared this predicted curve for the prospective dataset *D*_*P*_ and the Kaplan–Meier survival curve^15^ *P*_*P*_(*t*) directly derived from *D*_*P*_.

### Statistical Analysis

Normally distributed data were expressed as mean ± standard deviation; other values were reported as a median and interquartile range (IQR). We conducted the goodness-of-fit test and used the coefficient of determination as a measure to assess the significant relationships between the predictive curves and actual Kaplan–Meier curves of the cardiovascular event-free rate. The differences in the predictive curves were tested using the Wilcoxon signed-rank test. We estimated the error bounds of the parameters, *α* and *β*, by applying the standard bootstrap sampling^16^. All tests were two-tailed, and *P* < 0·05 was considered significant. All analyses were performed using the JMP software for Windows (version 8.0.2, SAS Inc., Cary, NC).

## Results

### Patients characteristics

In the retrospective study (Protocol I), the clinical characteristics of the patients are summarized in Table 1. In 78 patients, cardiovascular events (*n* = 71 for HF rehospitalization, and *n* = 14 for HF-related death) occurred at a median time of 260 days after discharge and the remaining 89 patients had no cardiovascular events by a median time of 859 days after discharge (range, 515–1194 days). Among clinical parameters, we selected the highest coefficient values of 50 parameters without the data of cardiac catheterization; the 50 clinical parameters with coefficient values for constructing the mathematical formula are depicted in Table 2. In the prospective study (Protocol II), the clinical characteristics of 213 patients are summarized in Table 3. Of these, 84 patients were readmitted to each hospital at a median time of 161 days after discharge, and 21 patients died due to worsening of HF at a median time of 275 days; the remaining 114 patients had no cardiovascular events by a median time of 636 days after discharge (range, 183–898 days).

**Table 1:** Patient characteristics in the retrospective study.

Clinical factors	Population (n = 167)
Age, (years)	72 (60–79)
Gender, Male/Female	98/69
**Signs at admission**	
NYHA class (II/III/IV)	52/54/61
Elevated jugular venous pressure	97 (58)
S3 gallop	107 (64)
Leg edema	91 (54)
**Cause of ADHF**	
Cardiomyopathy	56 (34)
Hypertensive heart disease	25 (15)
Ischemic heart disease	16 (10)
Valvular heart disease	47 (28)
Cardiac sarcoidosis	7 (4)
Myocarditis	1 (1)
Others	15 (9)
**Comorbidity**	
Hypertension	81 (49)
Diabetes mellitus	39 (23)
Hyperlipidemia	47 (28)
Chronic Af	67 (40)
Cerebrovascular disease	31 (19)
Obstructive pulmonary disease	10 (6)
**Baseline characteristics at admission/at discharge**	
Heart rate (beats/min)	81(69–103)/68 (60–76)
Systolic BP (mmHg)	126(108–152)/110 (100–120)
Diastolic BP (mmHg)	70 (60–88)/60 (56–70)
Body weight (kg)	58 (49–68)/53 (44–61)
**Laboratory factors at discharge**	
Hemoglobin, (g/dl)	12.0 (10.7–13.4)
WBC, (/μl)	5600 (4600–6600)
Albumin, (g/dl)	3.9 (3.6–4.1)
AST, (U/l)	25.0 (20.5–31.5)
ALT, (U/l)	19.0 (13.0–18.0)
BUN, (mg/dl)	21.0 (16.0–30.8)
Creatinine, (mg/dl)	0.9 (0.8–1.3)
Sodium, (mEq/l)	138 (135–140)
Uric acid, (mg/dl)	7.0 (5.7–8.4)
CRP, (mg/dl)	0.18 (0.04–0.53)
HbA1C, (%)	5.5 (5.2–6.2)
BNP, (pg/ml)	191 (102–413)
**Echocardiographic factors at admission/at discharge**	
LVDd (mm)	59 (49–66)/58 (49–66)
LVDs (mm)	48 (36–57)/45 (34–58)
%FS (%)	19 (11–29)/20 (13–31)
IVST (mm)	9 (8–11)/9 (8–11)
PWT (mm)	10 (8–11)/10 (8–11)
LAD (mm)	49 (43–57)/45 (39–54)
Pressure across tricuspid valve (mmHg)	34 (25–42)/22 (14–27)
**Medication at admission**	
Use of dopamine	6 (4)
Use of dobutamine	35 (21)
Use of phosphodiesterase inhibitor	28 (17)
Use of hANP	38 (23)
Use of nitroglycerin	27 (16)
Use of intravenous diuretic	76 (46)
**Oral medications at discharge**	
ACE inhibitor	80 (48)
ARB	35 (21)
**Beta-blockers**	
Digitalis	48 (29)
Diuretics	151(90)

Data are given as the median (interquartile range) or n (%). ACE inhibitor, angiotensin-converting enzyme inhibitor; ADHF, acute decompensated heart failure; Af, atrial fibrillation; ARB, angiotensinII receptor blocker; BNP, B-type natriuretic peptide; BUN, Blood urea nitrogen; BP, blood pressure; CRP, C-reactive protein; FS, fractional shortening; hANP, human atrial natriuretic peptide; IVST, interventricular septum thickness; LAD, left atrial dimension; LVDd, Left ventricular end-diastolic dimension; LVDs, Left ventricular end-systolic dimension; NYHA, New York Heart Association; PWT, posterior wall thickness.

**Table 2:** The 50 clinical parameters with coefficient values for constructing the mathematical formula.

Clinical factors			Maximum Values	coefficient values
Age, (years)	72	(60–79)	91	1.789
Gender, Male/Female	98/69		1	0.362
NYHA class (II/III/IV) at admission	52/54/61		4	0.022
Heart rate at admission (beats/min)	81	(69–104)	142	0.539
Leg edema	91	(54)	1	−0.692
**Cause of ADHF**				
Cardiomyopathy	56	(34)	1	−0.439
Hypertensive heart disease	25	(15)	1	−0.363
Ischemic heart disease	16	(10)	1	10.842
Valvular heart disease	47	(28)	1	−0.282
**Comorbidity**				
Hypertension	81	(49)	1	0.134
Hyperlipidemia	47	(28)	1	0.288
Chronic Af	67	(40)	1	−0.185
Cerebrovascular disease	31	(19)	1	−0.108
Obstructive pulmonary disease	10	(6)	1	−0.604
CRT	35	(20)	1	−0.719
ICD	35	(20)	1	−0.554
Pacemaker	14	(8)	1	0.752
Number of family members	1	(1–2)	6	−0.525
Albumin at admission, (g/dl)	3.7	(3.4–4.0)	4.9	−1.773
CRP at admission, (mg/dl)	0.3	(0.1–0.9)	24.5	−1.000
LVDs at admission, (mm)	48	(36–57)	79	−0.085
%FS at admission, (%)	19	(11–29)	65	0.567
WBC at admission, (/μl)	6500	(5000–8850)	23500	0.661
AST at discharge, (U/l)	25.0	(20.5–21.5)	575	−3.374
BUN at discharge, (mg/dl)	21.0	(16–30.8)	133	0.772
Uric acid at discharge, (mg/dl)	7.0	(5.7–8.4)	13.1	−0.051
CRP at discharge, (mg/dl)	0.18	(0.04–0.53)	17.22	−1.265
BNP at discharge, (pg/ml)	191	(102–413)	3257	−0.826
%FS at discharge, (%)	20	(13–31)	53	−1.013
IVST at discharge, (mm)	9	(8–11)	17	−0.889
AR grade (≥II) at discharge	21	(13)	3	−0.422
MR grade (≥II) at discharge	48	(29)	4	0.090
TR grade (≥II) at discharge	43	(26)	4	0.379
**Oral medications at discharge**				
ACE inhibitor	80	(48)	1	0.017
Anti-allergic	12	(7)	1	−0.335
Anti-inflammatory drug	5	(3)	1	−0.563
Antiplatelet	45	(27)	1	0.252
Antithyroid drug	2	(1)	1	−1.751
Beta-blockers	109	(65)	1	0.720
Bronchodilator	7	(4)	1	−0.489
Cardiotonic drug	22	(13)	1	−0.466
Choleretic drug	10	(6)	1	−0.847
Digitalis	48	(29)	1	0.194
Diuretics	151	(90)	1	0.971
Intestinal disease drug	4	(2)	1	−0.163
Lipid-lowering drug	37	(22)	1	0.307
Proton pump inhibitor	60	(36)	1	−0.023
Purgative	49	(29)	1	0.279
Sedative-hypnotic (benzodiazepin)	36	(22)	1	−0.392
Vitamins	14	(8)	1	0.211
(Constant)				0.9014

Data are given as the Median (interquartile range) or n (%). ACE inhibitor, angiotensin-converting enzyme inhibitor; ADHF, acute decompensated heart failure; Af, atrial fibrillation; AR, aortic regurgitation; BNP, B-type natriuretic peptide; BUN, Blood urea nitrogen; CRT, cardiac resynchronization therapy; CRP, C-reactive protein; FS, fractional shortening; ICD, Implantable Cardioverter Defibrillator; VST, interventricular septum thickness; LVDs, Left ventricular end-systolic dimension MR, mitral regurgitation; NYHA, New York Heart Association; TR, tricuspid regurgitation.

**Table 3:** The selected 50 clinical parameters in the prospective study.

Clinical factors		
Age, (years)	71	(60–79)
Gender, Male/Female	135/78	
NYHA class (II/III/IV) at admission	47/108/58	
Heart rate at admission (beats/min)	80	(68–97)
Leg edema	136	(64)
**Cause of ADHF**		
Cardiomyopathy	76	(36)
Hypertensive heart disease	29	(14)
Ischemic heart disease	26	(12)
Valvular heart disease	47	(22)
**Comorbidity**		
Hypertension	108	(51)
Hyperlipidemia	73	(34)
Chronic Af	104	(49)
Cerebrovascular disease	14	(7)
Obstructive pulmonary disease	7	(3)
CRT	24	(11)
ICD	31	(15)
Pacemaker	25	(12)
Number of family members in the same household	1	(1–2)
Albumin at admission, (g/dl)	3.8	(3.5–4.1)
CRP at admission, (mg/dl)	0.4	(0.1–1.1)
LVDs at admission, (mm)	47	(34–58)
%FS at admission, (%)	18	(10.3–30.6)
WBC at admission, (/μl)	5240	(4200–6400)
AST at discharge, (U/l)	23	(18–30)
BUN at discharge, (mg/dl)	23	(18–35)
Uric acid at discharge, (mg/dl)	6.5	(5.3–7.9)
CRP at discharge, (mg/dl)	0.2	(0.1–0.6)
BNP at discharge, (pg/ml)	244.8	(117–457)
%FS at discharge, (%)	20.9	(11–31.7)
IVST at discharge, (mm)	10	(8–11)
AR grade (≥II) at discharge	26	(12)
MR grade (≥II) at discharge	93	(44)
TR grade (≥II) at discharge	59	(28)
**Oral medications at discharge**		
ACE inhibitor	111	(52)
Anti-allergic	8	(4)
Anti-inflammatory drug	48	(23)
Antiplatelet	27	(13)
Antithyroid drug	3	(1)
Beta-blockers	161	(76)
Bronchodilator	2	(1)
Cardiotonic drug	36	(17)
Choleretic drug	11	(5)
Digitalis	42	(20)
Diuretics	181	(85)
Intestinal disease drug	19	(9)
Lipid-lowering drug	79	(37)
Proton pump inhibitor	119	(56)
Purgative	63	(30)
Sedative-hypnotic (benzodiazepin)	12	(6)
Vitamins	7	(3)

Data are given as the median (interquartile range) or n (%). ACE inhibitor, angiotensin-converting enzyme inhibitor; ADHF, acute decompensated heart failure; Af, atrial fibrillation; AR, aortic regurgitation; BNP, B-type natriuretic peptide; BUN, Blood urea nitrogen; CRT, cardiac resynchronization therapy; CRP, C-reactive protein; FS, fractional shortening; ICD, Implantable Cardioverter Defibrillator; VST, interventricular septum thickness; LVDs, Left ventricular end-systolic dimension MR, mitral regurgitation; NYHA, New York Heart Association; TR, tricuspid regurgitation.

### Predictive capability of the mathematical formula for the prospective outcomes

**Figure 1:**
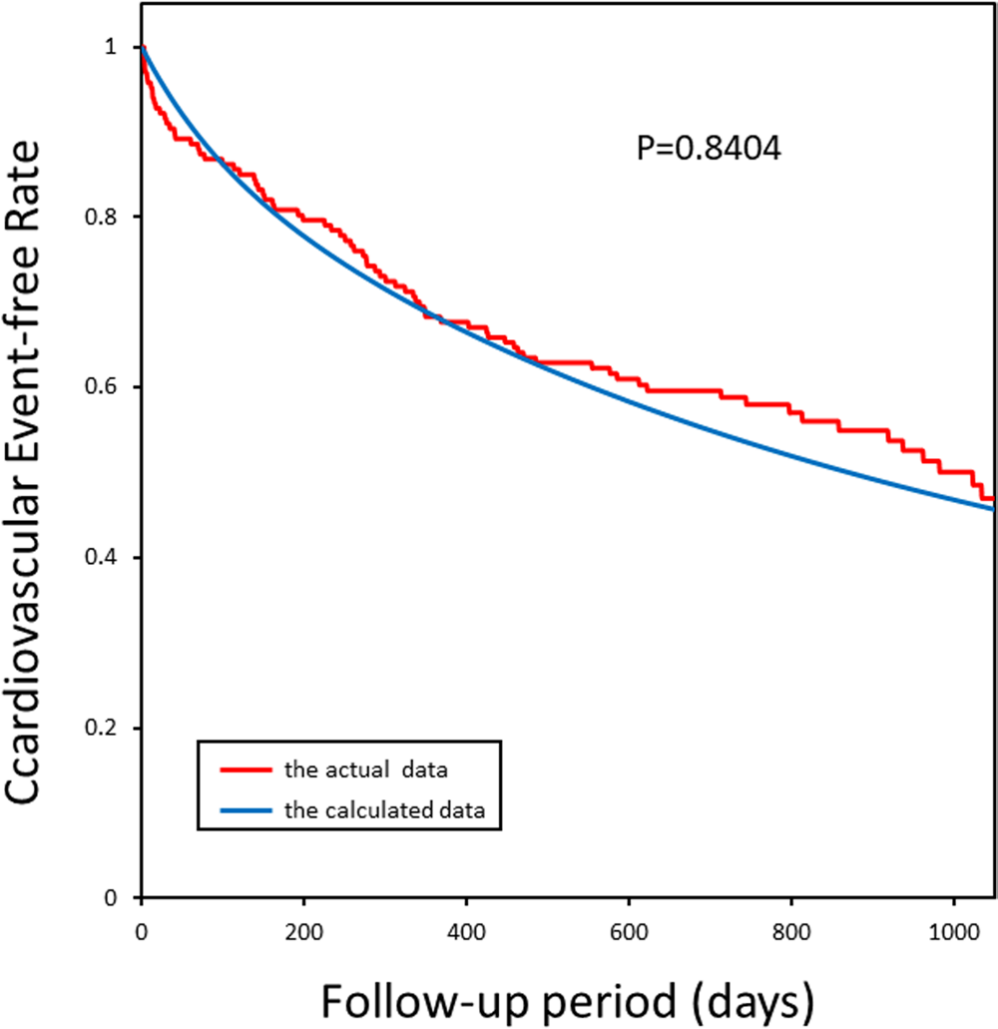
The Kaplan-Meier plots of calculated and actual cardiovascular event-free rates in Protocol I (the retrospective study). The actual cardiovascular events started slightly later than the calculated events and ended earlier than the calculated events; however, the goodness-of-fit model found that KM and predictive curves were significantly close, and the coefficient of determination was P = 0.8404.

**Figure 2:**
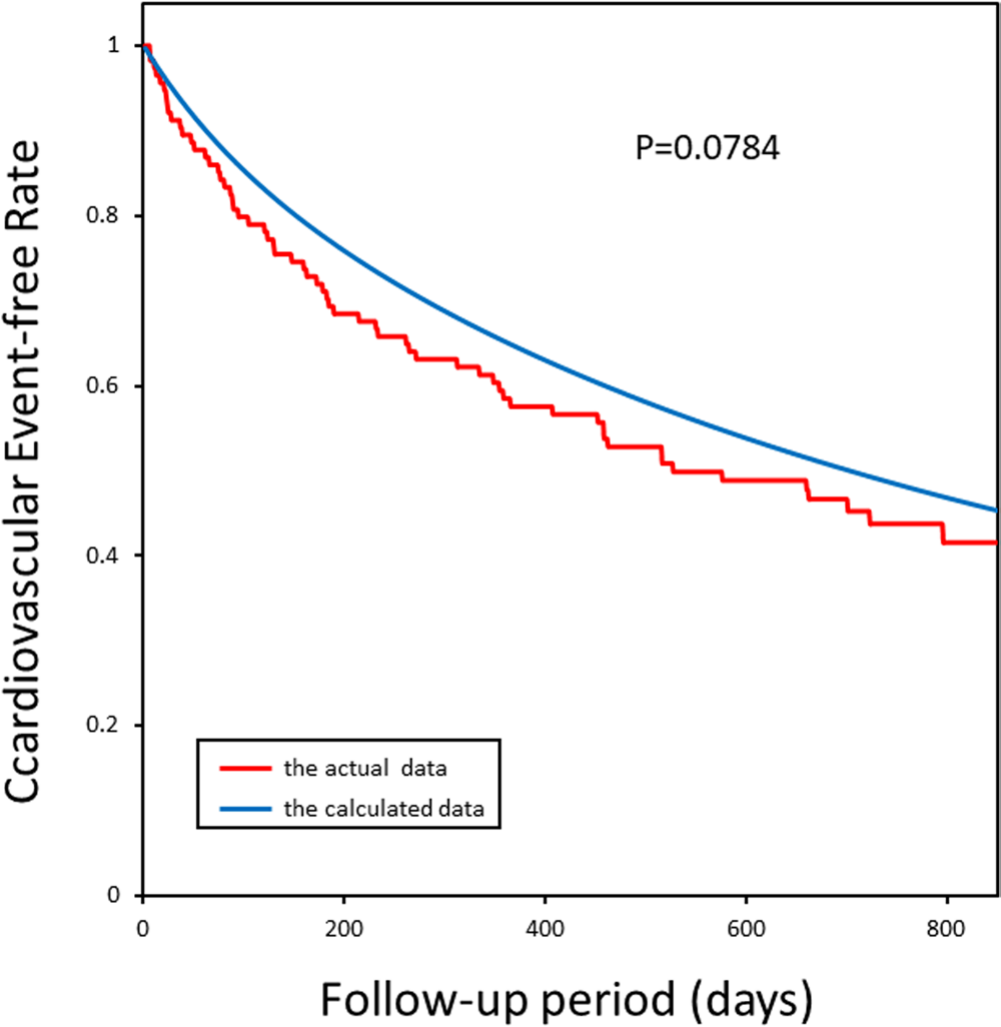
The Kaplan-Meier plots of calculated and actual cardiovascular event-free rates in patients in NCVC in Protocol II (the prospective study). The actual cardiovascular events started slightly later than the calculated events and ended earlier than the calculated events; however, the goodness-of-fit model found that KM and predictive curves were significantly close, and the coefficient of determination was P = 0.0784.

**Figure 3:**
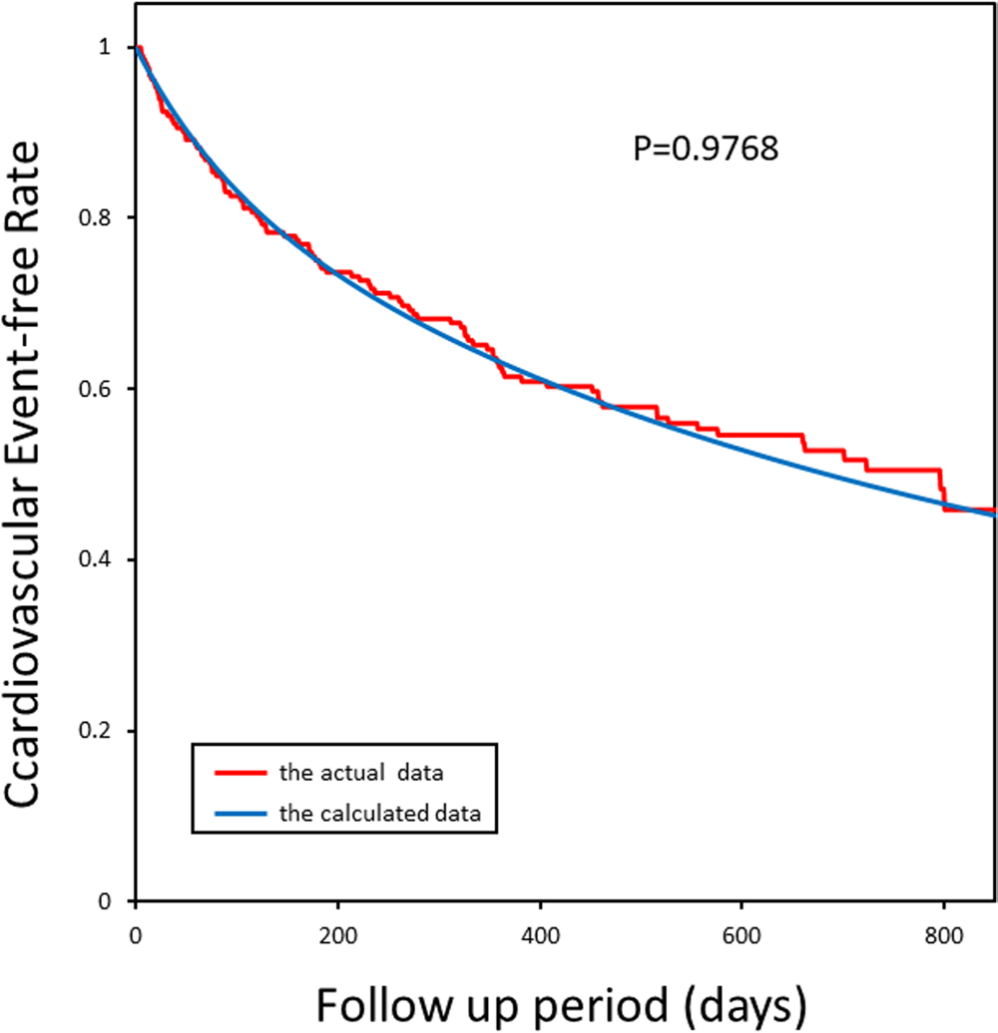
The Kaplan-Meier plots of calculated and actual cardiovascular event-free rates in patients in NCVC in Protocol II (the prospective study). The goodness-of-fit model found that KM and predictive curves were significantly close, and the coefficient of determination was P = 0.9768.

We confirmed that the Kaplan–Meier curves using this formula and actual data in the retrospective study revealed the proper fitting of the probability of cardiovascular outcomes (Fig. 1). Then, in the prospective study, we just analyzed the prospective data using only our institute. Figure 2 shows that the mathematical formula obtained from the retrospective study can predict the clinical outcomes observed in the prospective study. Thus, we tested whether our formula can predict the probability of cardiovascular events in all the institutes, and we found that our formula can predict the clinical outcomes for three institutes (Fig. 3).

### The factors that provoke or prevent cardiovascular events in 50 clinical factors

Since we found that the mathematical formula applies to predict the occurrence of cardiovascular events in the prospective study, we assumed that each attribute coefficient for this mathematical formula is also essential for the clinical practice for HF (Table 2). When we investigated the contribution of each parameter for the objective measure, we found that ischemic heart disease results in a worse prognosis. In the physical examination, high heart rate or implantation of pacemaker classification was the worse factor, and the implantation of cardiac resynchronization therapy or implantable cardioverter defibrillator demonstrated better outcomes. Furthermore, the data of blood analysis, echocardiography and oral medications related to the cardiovascular events in the complex and confounding manners. Intriguingly, the number of family members resulted in a better prognosis.

## Discussion

This study provided the evidence that the mathematical formula using the retrospective clinical data provides the occurrence probability of cardiovascular events in the prospective study in patients with HF. We were able to derive the formula of *α* = *f*(*x*_1_, …, *x*_50_), where *α* is the probability of the cardiovascular events and *x*_1_, …, *x*_50_ are clinical factors observed before cardiovascular events, which could prospectively predict the occurrence probability of cardiovascular events. This study proposes the novel idea that the occurrence probability of future cardiovascular events can be mathematically formulated and deduced from the retrospective clinical and personal parameters before the time of cardiovascular events.

Importantly, we found that the occurrence probability depends not only on the cardiac dysfunction but also the dysfunction parameters of other organs, such as the kidneys and liver, and social factors, such as the number of family members living with a patient. Therefore, we can regard the occurrence probability as the overall severity of HF. This concept is well matched to the idea that we need to investigate the effect of certain treatment of HF by judging the mortality or morbidity, but not by cardiac function in large-scale clinical trials^18^. The mortality or morbidity during a certain observation period is depicted by the Kaplan–Meier curves, which represent the occurrence probability of cardiovascular events.

What is the differences between the present and previous studies to assess clinical outcomes? The earlier studies, including ours^19–21^, have merely identified the important factors for cardiovascular outcomes using the cohort data of patients with HF. In such studies, clinical data are retrospectively or prospectively collected and identified the most influential factors using the multivariate analysis. However, no researcher has tested whether such multiple factors can quantitatively predict the occurrence probability of future cardiovascular events. Most of all, arbitrary factors, which are unintentionally collected by investigators and usually ignored, may be essential factors to explain the occurrence probability, and the investigator-intended analysis of the data cannot cover such arbitrary factors beyond expectation. This is the concept of analysis of big data or data mining analysis^22^. Wang *et al*.^23^ revealed that although multiple biomarkers are associated with a high relative risk of adverse events, even the combination of these factors only moderately improved the prediction of risk in an individual. This suggests that the occurrence of cardiovascular events may not be well predictable even after the multiple factors are convoluted. In contrast, we collected almost all the numerical data in the medical records documented before the onset of cardiovascular events and solved the mathematical formula using these parameters to provide the exact probability for future cardiovascular events. Of more than 250 clinical factors that constitute the original mathematical formula^8^, we selected the 50 most influential factors and re-solved the mathematical formula. The mathematical formula using these 50 factors potentially validates its plausibility for the calculation of the occurrence probability of cardiovascular events in patients with HF, suggesting that we need more clinical data to predict the future outcomes or obtain the mathematical formula for the prediction than we expected. WBC values at admission may approximately indicate the unique value of each patient. On the other hand, the most abnormal values at the admission may determine the severity of the pathophysiology of CHF.

How do we interpret the mathematical formulae given in the present study? One may argue that our process is just adjusting or fitting the clinical data with the clinical outcomes using the mathematical formula. Nevertheless, if the clinical parameters had no relation to the time of the occurrence of cardiovascular events, we could not have fitted clinical parameters with the objective measures. Since we could fit the clinical parameters before the time of the occurrence of cardiovascular events with the objective function of the probability of cardiovascular events, we consider that our fitting process of the mathematical formula seems reasonable. To further confirm the feasibility and applicability of the framework of the present investigation, we agreed to this criticism against our previous work^8^ and decided to perform the prospective study to test the validity of our mathematical formula to predict the possibility of future cardiovascular events. Figures 2 and 3 support our hypothesis; thus, we can propose the predictability and reproducibility of the occurrence of cardiovascular events in patients with HF using the mathematical models. On the other hand, the patients’ characteristics for retrospective and prospective studies are quite different, as shown in Tables 2 and 3. Patients for the prospective study seemed to have suffered from severer HF than those for the retrospective study. Nevertheless, the Kaplan–Meier curves produced by the formula can provide the right fitting for the actual data of the prospective study, suggesting that the present formula is valid for any group of patients with HF.

It would be intriguing to see the coefficient of each clinical parameter for the mathematical formula. We have to note that we revealed that 50 factors are essential to constitute the function of the occurrence probability of cardiovascular events, however, these factors are confounded in each other, of which the mathematical formula is created, indicating that we should recognize the importance of the network of these 50 factors in creating the formula rather than the clinical impact of each factor. We should be cautious of the fact that some of the 50 clinical parameters are largely and sensitively affected by the acute changes of the pathophysiology of HF. Since such parameters contribute to the creation of the present formula, we can only conclude that each value at the admission or the discharge in each patient affects the occurrence probability of cardiovascular events after discharge. We need to investigate the pathophysiological meaning in the future study.

The most important issue is that we can provide the predictive model of cardiovascular events in HF patients using 50 factors and verify the feasibility of the model in the cohort of HF patients in 3 different institutes.

Another important point of this study is that we formed the mathematical formula by the retrospective clinical data in National Cerebral and Cardiovascular Center at the central part of Japan and tested the applicability in the prospective data in Hokkaido University located in the north of Japan and Kyushu University at the southern part. Although one may consider that this mathematical formula is only valid in National Cerebral and Cardiovascular Center, it is not the case. In fact, this mathematical formula to predict the possibility of cardiovascular events in patients with HF is valid throughout Japan. This mathematical formula may not be valid in other countries; however, the pathophysiology and treatment strategy of HF are common worldwide, suggesting that such formulas should be valid to provide the future occurrence of cardiovascular events in other countries. Of course, the concept to create a mathematical formula should be translated and transmitted worldwide to know the real risk of cardiovascular events and to treat the clinical factors using their data in patients with HF.

There are several applications and limitations for the present study. First of all, since these 50 clinical parameters can be easily provided in outpatient or inpatient clinics, we can evaluate the severity of HF from the viewpoint of the probability of the onset of cardiovascular events in each patient. Secondly, we can identify what clinical factors increase the probability of cardiovascular events, suggesting that we can identify the target of the treatment of HF in each patient. Thirdly, this formula may provide the educational tool for the HF patients. Fourthly, the concept of the creation of formula to predict the clinical outcomes may be applicable to the other fields such as cerebral infarction or cancers^24^. On the other hand, we have some limitations of the present formula because we created the formula using the data of the HF patients with mild to moderate severity of HF symptom. Therefore, we are not able to apply the present formula to the severe HF patients to predict the occurrence probability of the cardiovascular events because we did not derive the present equation from the cohort of severe HF patients. To respond this requirement, we need to create the mathematical formula using the data of the severe HF patients.

## Conclusions

We created a mathematical formula that precisely provides the probability of the clinical outcomes of patients who are hospitalized with ADHF and discharged after appropriate treatment. Mathematics using the present cardiovascular big data may predict the occurrence probability of future cardiovascular events. Since we found the importance of the clinical parameters independent of cardiac function, it merits the better treatment of HF.
